# *Bacillus anthracis *spore interactions with mammalian cells: Relationship between germination state and the outcome of *in vitro*

**DOI:** 10.1186/1471-2180-11-46

**Published:** 2011-02-28

**Authors:** Ian M Gut, Batcha Tamilselvam, Angela M Prouty, Bojana Stojkovic, Stephanie Czeschin, Wilfred A van der Donk, Steven R Blanke

**Affiliations:** 1 Department of Microbiology, University of Illinois, Urbana, IL, 61801, USA; 2 Department of Chemistry and Howard Hughes Medical Institute, University of Illinois, Urbana, IL, 61801, USA; 3 Institute for Genomic Biology, University of Illinois, Urbana, IL, 61801, USA; 4 Naval Medical Research Center, Biological Research Directorate, BDRD Annex, Rockville, MD, 20852, USA; 5 Parkland College, Champaign, IL, 61821, USA

## Abstract

**Background:**

During inhalational anthrax, internalization of *Bacillus anthracis *spores by host cells within the lung is believed to be a key step for initiating the transition from the localized to disseminated stages of infection. Despite compelling *in vivo *evidence that spores remain dormant within the bronchioalveolar spaces of the lungs, and germinate only after uptake into host cells, most *in vitro *studies of infection have been conducted under conditions that promote rapid germination of spores within the culture medium.

**Results:**

Using an *in vitro *model of infection, we evaluated the influence of the germination state of *B. anthracis *spores, as controlled by defined culture conditions, on the outcome of infection. Spores prepared from *B. anthracis *Sterne 7702 germinated in a variety of common cell culture media supplemented with fetal bovine serum (FBS) while, in the absence of FBS, germination was strictly dependent on medium composition. RAW264.7 macrophage-like cells internalized spores to the same extent in either germinating or non-germinating media. However, significantly more viable, intracellular *B. anthracis *were recovered from cells infected under non-germinating conditions compared to germinating conditions. At the same time, RAW264.7 cells demonstrated a significant loss in viability when infected under non-germinating conditions.

**Conclusions:**

These results suggest that the outcome of host cell infection is sensitive to the germination state of spores at the time of uptake. Moreover, this study demonstrates the efficacy of studying *B. anthracis *spore infection of host cells within a defined, non-germinating, *in vitro *environment.

## Background

Inhalational anthrax commences with the deposition of *Bacillus anthracis *spores into the bronchioalveolar spaces of the lungs, and culminates with the systemic dissemination of vegetative bacilli within the host [1-3]. Within the lungs, internalization of dormant spores, possibly by several different types of host cells, is believed to be a key step for initiating the transition from the localized to disseminated stages of infection. Alveolar macrophages are reported to transport spores out of the lungs to regional lymph nodes [4-7]. Dendritic cells have also been implicated in the rapid carriage of spores to the draining lymph nodes [8,9]. Finally, alveolar epithelial cells have recently been demonstrated to internalize spores both *in vitro *and *in vivo *[10-12], and have been proposed to facilitate the transcytosis of *B. anthracis *across the epithelial barrier. Taken together, these findings suggest that *B. anthracis *may escape the lungs by several distinct mechanisms.

To characterize the interaction of *B. anthracis *spores with host cells during the early stages of inhalational anthrax, *in vitro *models of infection have been widely implemented [8,13-22]. The tractability of *in vitro *models has facilitated new insights into the molecular and cellular basis of spore binding and uptake, as well as host cell responses. Nonetheless, the use of *in vitro *models has resulted in a striking lack of consensus as to the responses and fates of both intracellular *B. anthracis *and infected cells. Although there are multiple reports of germinated spores within host cells [13,15,16,20,23], several studies have indicated that germinated spores ultimately kill macrophages [13,19,20], while others have reported that macrophages readily kill intracellular *B. anthracis *[21,22]. The lack of consensus may be due, in part, to fundamental differences between the infection models used by research groups, which includes variability in bacterial strains, mammalian cells, and experimental conditions employed.

An important issue that is likely to directly influence the outcome of *in vitro *models of infection is the germination state of spores as they are internalized into host cells. Several *in vivo *lines of evidence support the idea that spores remain dormant in the alveolar spaces of the lungs prior to uptake. First, dormant spores have been recovered from the lungs of animals several months after initial infection [7,24]. Second, all spores collected from the bronchial alveolar fluids of spore-infected Balb/c mice were found to be dormant [5,23]. In contrast, a substantial percentage of intracellular spores recovered from alveolar macrophages were germinated [23]. Third, real time *in vivo *imaging failed to detect germinated spores within lungs, despite the effective delivery of dormant spores to these organs [25-27]. One of these studies [25] reported that vegetative bacteria detected in the lungs during disseminated *B. anthracis *infection arrived at the lungs via the bloodstream, rather than originating from *in situ *spore growth. Finally, using spores that had been engineered to emit a bioluminescent signal immediately after germination initiation, a recent study reported that germination was commenced in a mouse model of infection only after spore uptake into alveolar macrophages [6]. However, despite considerable evidence that the lung environment is not intrinsically germinating for *B. anthracis *spores, most *in vitro *infection models have been conducted using culture media containing FBS and/or specific L-amino acids or nucleotides at concentrations previously demonstrated to promote germination of spores *in vitro *[20,28-34]. Under such conditions, it is likely that, in these previous studies, host cells were infected with heterogeneous populations of germinated and dormant spores.

The objective of this study was to experimentally address existing gaps in knowledge as to how the germination state of *B. anthracis *spores, as dictated by the presence or absence of serum during *in vitro *infections, influences the uptake of spores into mammalian cells, as well as the subsequent fate of both intracellular *B. anthracis *and infected cells. Germinating and non-germinating culture conditions were used to compare the interaction of spores prepared from *B. anthracis *Sterne 7702 with RAW264.7 macrophage-like cells, as well as several other cell lines. These studies revealed that the uptake of *B. anthracis *into cells was largely unaffected by the germination state of spores. In contrast, the number of viable, intracellular *B. anthracis *recovered from infected cells, as well as the viability of the infected cells, was dependent on the germination state of spores during uptake. These results support the idea that the germination state of spores is an important consideration when interpreting results from *in vitro *infections with *B. anthracis *spores.

## Results and Discussion

### The composition of cell culture medium influences the germination and outgrowth of *B. anthracis *spores

**Figure 1:**
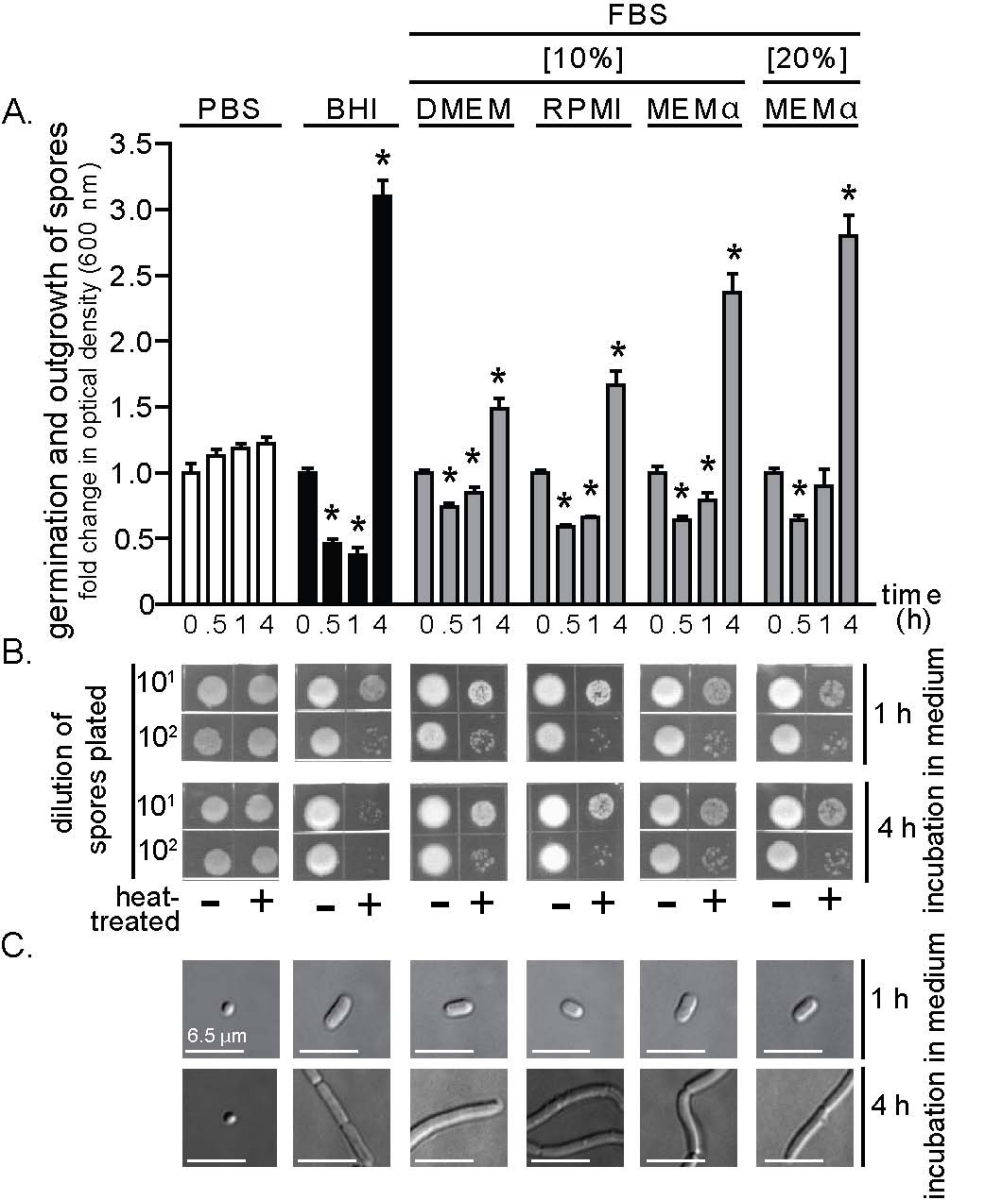
**FBS in cell culture media promotes germination and outgrowth of *B. anthracis *spores**. *B. anthracis *spores were incubated in 96-well plates at 37°C and with rotary agitation in the indicated medium. Germination and outgrowth of spores were monitored at the indicated times. Medium conditions are listed at the top of the figure, and applicable to (A-C). (A) Optical determination of germination and outgrowth. The data are rendered as the O.D._600 nm _of the spore suspension at the indicated times relative to the original O.D._600 nm _of the spore suspension at time = 0 of the 37°C incubation. For BHI, DMEM, RPMI, and MEMα, initial decreases in O.D._600 nm _reflect the loss of spore refractility that occurs subsequent to germination initiation, while the increases in O.D._600 nm _measured at later time points (1 and 4 h) reflects bacterial replication. For PBS, the modest increases in O.D._600 nm _are due to time-dependent medium evaporation. Error bars indicate standard deviations. For each medium tested, the *P*-values were calculated to evaluate the statistical significance of the differences between O.D._600 nm _values at the indicated times and O.D._600 nm _values at the initial time point. (B) Spore heat sensitivity as a function of medium conditions. Aliquots from spore cultures were removed at indicated times, incubated for 30 min at either at 65°C or on ice, diluted 10^1^- or 10^2^-fold (PBS pH 7.2), spotted (10 μL) on LB plates, and incubated at 25°C. After 18 h, the plates were photographed. (C) Visual determination of *B. anthracis *spore outgrowth as a function of cell culture medium. Aliquots from spore cultures were removed at indicated times and analyzed for outgrowth using DIC microscopy. The bars indicate a length of 6.5 μm. The data in (A) are combined from 3 independent experiments. The data in (B) and (C) are from a single experiment, and are representative of 3 independent experiments.

Several commonly used mammalian cell culture media, in the presence or absence of fetal bovine serum (FBS), were first evaluated for the capacity to induce germination initiation, which is the earliest set of changes in dormant spores triggered by the presence of germinants. Spore outgrowth, which is the transition of germinated spores into vegetative bacilli [35-37], was also evaluated. These studies revealed that, regardless of the medium tested, dormant spores prepared from *B. anthracis *Sterne 7702 (1.0 × 10^8 ^spores/mL) underwent germination initiation when incubated at 37°C and under 5% CO_2 _in the presence of FBS, as indicated by increased sensitivity of the spores to heat treatment [38] and a time-dependent decrease in spore refractility, which indicates rehydration of the spore core following germination initiation [39] (Table 1, Figure 1A, B). When incubated in Dulbecco's modified Eagle's medium (DMEM) plus 10% FBS, or, Roswell Park Memorial Institute (RPMI) 1640 medium plus 10% FBS, 86.0 ± 5.2% and 83.4 ± 2.6% of total spores, respectively, converted from heat-resistant to heat sensitive forms within 10 min, while 97.6 ± 0.2% and 96.6 ± 2.2% of total spores, respectively, converted to heat sensitive forms within 60 min, as determined by dilution plating and direct CFU counting over the course of three independent experiments. These results are consistent with a previous study reporting that approximately 98% of the *B. anthracis *Sterne spores germinated within an hour when incubated in DMEM plus 10% FBS [13,20]. Another previous study reported that when incubated in minimal essential medium (MEM) supplemented with 10% FBS, approximately 37% of Sterne spores germinated within one hour [40]. Dose response studies revealed that germination initiation was induced in DMEM containing 1% FBS, but not 0.5% FBS (Table 2). Spore germination or outgrowth was not dependent on the commercial source of FBS, as similar results were obtained with FBS purchased from 3 different vendors (data not shown). The capacity of spore preparations to germinate were confirmed by incubating dormant spores in the presence of the known germinants, L-alanine and L-inosine (each at 10 mM, in phosphate buffered saline (PBS) pH 7.2) (Table 1). In addition, the capacity of spore preparations to germinate and outgrow were confirmed by incubating dormant spores in the presence of Luria-Bertani broth (LB) (Table 1), as previously reported [41-43]. The time dependent increase in culture density (Figure 1A) and morphological conversion of spores into elongated bacilli (Figure 1C) indicated that in medium containing FBS, there was outgrowth of spores into vegetative bacilli.

**Table 1:** Germination and outgrowth of *B. anthracis *spores as a function of cell culture medium in the presence or absence of FBS*^a^*.

			outgrowth*^e^*	
			
medium*^b^*	FBS*^c^*	germination*^d^*	1 h	4 h
DMEM	-	-	-	-
	+	+	+	+
RPMI	-	-	-	-
	+	+	+	+
MEMα	-	+	+	+
	+	+	+	+
MEM	-	-	-	-
	+	+	+	+
AMEM	-	-	-	-
	+	+	+	+
EMEM	-	-	-	-
	+	+	+	+
BME	-	-	-	-
	+	+	+	+
CIM	-	+	+	+
	+	+	+	+
F-12	-	-	-	-
	+	+	+	+
M5A	-	+	+	+
	+	+	+	+
BHI	-	+	+	+
LB	-	+	+	+
AA*^f^*	-	+	-	-

*^a ^*Three independent experiments were performed with three different spore preparations, each conducted in triplicate.

*^b ^*Spores prepared from *B. anthracis *Sterne 7702 were incubated in the indicated medium.

*^c ^*Indicates the presence (+) or absence (-) of 10% FBS in the indicated medium.

*^d ^*Spores were scored positive (+) for germination if the OD_600 nm _of the suspended spores decreased by more than 10% after 30 min incubation in the indicated medium.

*^e ^*Using DIC microscopy, spores were scored positive (+) for outgrowth if the spores bodies were visibly larger at 1 h, and had developed into vegetative bacteria by 4 h.

*^f ^*AA refers to L-alanine and L-inosine (each at 10 mM, in PBS pH 7.2).

**Table 2:** Germination and outgrowth of *B. anthracis *spores as a function of FBS concentration*^a^*.

			outgrowth*^e^*	
			
medium*^b^*	FBS (%)*^c^*	germination*^d^*	1 h	4 h
DMEM	0.0	-	-	-
	0.1	-	-	-
	0.5	-	-	-
	1.0	+	-	+
	5.0	+	+	+
	10.0	+	+	+

*^a ^*Three independent experiments were performed with three different spore preparations, each conducted in triplicate.

*^b ^*Spores prepared from *B. anthracis *Sterne 7702 were incubated in DMEM.

*^c ^*Indicates the concentration of FBS used in the DMEM.

*^d ^*Spores were scored positive (+) for germination if the OD_600 nm _of the suspended spores decreased by more than 5% after 30 min incubation in the indicated medium.

*^e ^*Using DIC microscopy, spores were scored positive (+) for outgrowth if the spores bodies were visibly larger at 1 h, and had developed into vegetative bacteria by 4 h.

**Figure 2:**
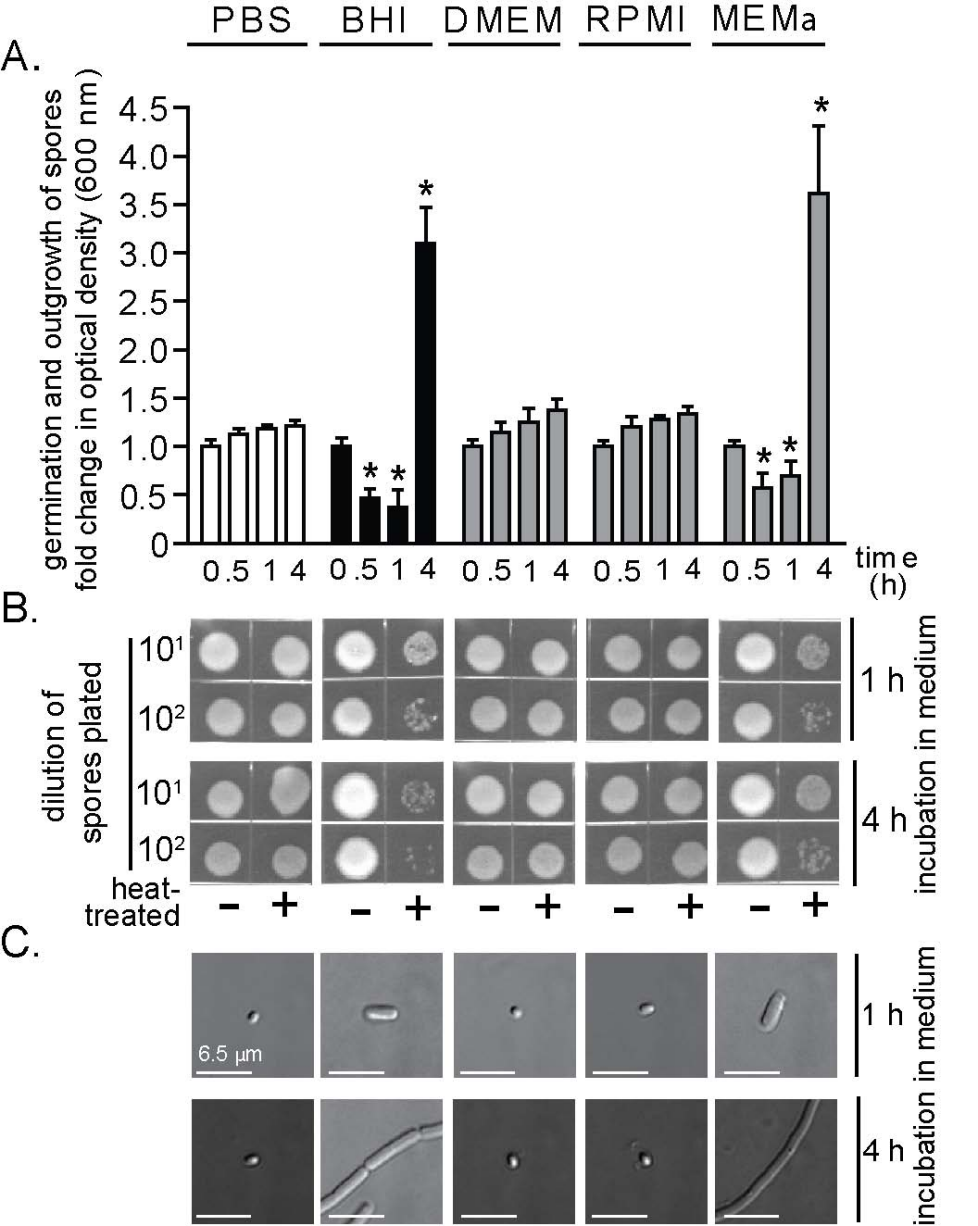
***B. anthracis *spore germination and outgrowth in FBS-free cell culture media**. *B. anthracis *spores were incubated in 96-well plates at 37°C and with rotary agitation within the indicated medium. Germination and outgrowth of spores were monitored at the indicated times. Medium conditions are listed at the top of the figure, and are applicable to (A-C). (A) Optical determination of germination and outgrowth. The data are rendered as the O.D._600 nm _of the spore suspension at the indicated times relative to the original O.D._600 nm _of the spore suspension at time = 0 of the 37°C incubation. Error bars indicate standard deviations. For each medium tested, the *P*-values were calculated to evaluate the statistical significance of the differences between O.D._600 nm _values at the indicated times and O.D._600 nm _values at the initial time point. (B) Spores heat sensitivity as a function of medium conditions. Aliquots from the spore cultures were removed at indicated times, incubated for 30 min at either at 65°C or on ice, diluted 10^1^- or 10^2^-fold (PBS pH 7.2), spotted (10 μL) on LB plates, and incubated at 25°C. After 18 h, the plates were photographed. (C) Visual determination of *B. anthracis *spore outgrowth as a function of cell culture medium. Aliquots from the spore cultures were removed at indicated times and analyzed for outgrowth using DIC microscopy. The bars indicate a length of 6.5 μm. The data in (A) are combined from 3 independent experiments. The data in (B) and (C) are from a single experiment and are representative of 3 independent experiments.

In the absence of FBS, several media were discovered to induce germination initiation and outgrowth of *B. anthracis *spores (Table 1). Germination initiation (30-60 min) and outgrowth were detected when spores were incubated in brain heart infusion (BHI) broth (Table 1, Figure 2), modified minimum essential medium alpha modification (MEMα) (Table 1, Figure 2), CO_2_-independent media (CIM) (Table 1), or McCoy's 5A (M5A) (Table 1). Each of these cell culture formulations contains all 20 amino acids, is enriched particularly in the known germinant L-alanine (15-20 mg/L), and also contains non-specified nucleotides. Notably, some nucleotides function as germinants [35,44,45]. In contrast, spores incubated in MEM, DMEM, RPMI 1640 medium, advanced MEM (AMEM), eagle MEM (EMEM), basal medium eagle (BME), or Ham's F-12 (F-12) did not germinate, even after 4 h (Figure 2, Table 2). Each of these media possesses lower concentrations of L-alanine (<10 mg/L) than those media that induced germination, and generally lacked nucleotides. These results emphasize that care must be exercised when selecting a culture medium for conducting *in vitro *infections under non-germinating conditions.

### Effects of pre-conditioned culture medium on the germination state of *B. anthracis *spores

**Figure 3:**
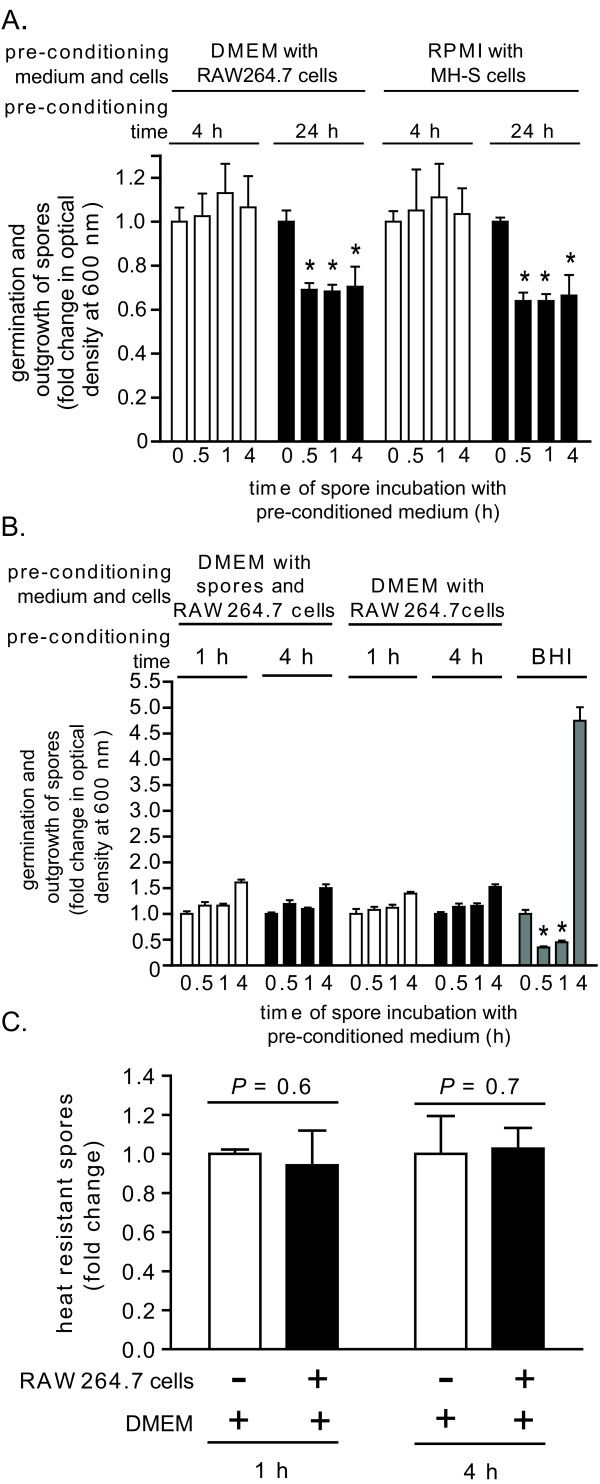
**The effects of pre-conditioned culture medium on the germination state of *B. anthracis *spores**. DMEM (A, B) or RPMI (B) were pre-conditioned by incubating with monolayers of RAW264.7 (A, B) or MH-S cells (B) at 37°C and under 5% CO_2_, in the absence (A) or presence (MOI 10) (B) of *B. anthracis *spores. (A, B). After 4 h (white bars) or 24 h (black bars) (A), or after 1 (white bars) and 4 h (black bars) (B), the medium was removed from the monolayers, filter sterilized, and then incubated with *B. anthracis *spores in 96-well plates at 37°C and with rotary agitation. Germination and outgrowth of spores were monitored at indicated times by measuring O.D._600 nm_. The results are rendered as the O.D._600 nm _of the spore suspension at the indicated time relative to the original O.D._600 nm _of the spore suspension at time = 0 of the 37°C incubation. *P*-values were calculated to evaluate the statistical significance of the differences between O.D._600 nm _values at the initial time point and O.D. O.D._600 nm _values at the indicated times. For (B), BHI (gray bars) was used as a positive control for germination and outgrowth. (C) An equal number of *B. anthracis *spores were incubated at 37°C and under 5% CO_2 _in DMEM (no FBS) in the absence (white bars) or presence (black bars) of RAW264.7 cells (MOI 10). At indicated times, aliquots of culture medium were removed, and spores were evaluated for heat resistance. The results are rendered as the number of heat resistant spores relative to spores incubated in DMEM alone, which were normalized to 1.0. *P*-values were calculated to evaluate the statistical significance of the differences in heat resistant spores between those incubated in the presence or absence of RAW264.7 cells. The data in (A-C) are combined from 3 independent experiments conducted in triplicate with error bars indicating standard deviations.

We next considered the possibility that cell culture media that normally do not promote spore germination may be converted to germinating media when incubated in the presence of mammalian cells. To evaluate this possibility, *B. anthracis *spores were incubated in DMEM or RPMI that had been "pre-conditioned" in the presence of RAW264.7 cells or MH-S cells, respectively. These studies revealed that neither DMEM nor RPMI, following a pre-conditioning period of 4 h, induced germination of *B. anthracis *spores (Figure 3A). Likewise, medium withdrawn from RAW264.7 cells infected for 1 or 4 h with dormant spores at a multiplicity of infection of 10 (MOI 10) also remained non-germinating (Figure 3B). Finally, medium withdrawn from RAW264.7 cells infected with dormant spores (MOI 10) contained only heat resistant *B. anthracis*, and no heat sensitive spores (Figure 3C), indicating that the extracellular spores remained dormant through the first 4 hours of infection. When the pre-conditioning period was extended to 24 h, both DMEM and RPMI induced germination, but negligible outgrowth, of spores (Figure 3A). Spore germination was eliminated by dialyzing (12-14 kDa molecular mass cutoff) the 24 h preconditioned DMEM or RPMI, but not by heat treatment (95°C for 10 min, or, 65°C for 30 min; data not shown), suggesting that the germinating factors were relatively small molecular weight, heat-resistant factors. Nonetheless, these studies confirm that *in vitro *models can be established that maintain a non-germinating environment for at least the first 4 h of infection.

### Mammalian cells remain viable and functional for at least 4 h in FBS-free culture medium

**Figure 4:**
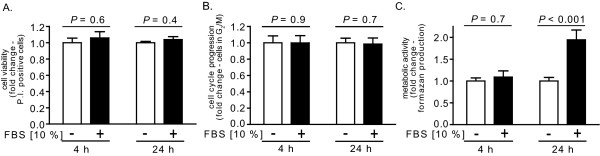
**Effect of non-germinating conditions on RAW264.7 cell viability, cell cycle progression, and metabolic activity**. RAW264.7 cells were incubated at 37° in DMEM in the presence (+, black bars) or absence (-, white bars) of FBS, and then evaluated at 4 or 24 h, as indicated, for viability (A), cell cycle progression (B), and metabolic activity (C). (A) The cells were assayed for PI uptake, as described under Materials and Methods. The data are rendered as the relative PI uptake normalized at both 4 and 24 h to cells incubated in the absence of FBS. (B) The cells were analyzed for their cell cycle profiles, as described under Materials and Methods. The data are rendered as the relative numbers of cells in G_2_/M normalized at both 4 and 24 h to cells incubated in the absence of FBS. (C) The cells were analyzed for conversion of MTT to formazan. The data are rendered as the fold change of formazan production normalized at both 4 and 24 h to cells incubated in the absence of FBS. To generate the bar graphs, data were combined from three independent experiments, each conducted in triplicate. Error bars indicate standard deviations. The *P *values were calculated to evaluate the statistical significance of the differences in viability (A), cell cycle progression (B), and metabolism (C) between cells cultured in the absence or presence of FBS.

Although a non-germinating environment was maintained for at least 4 h in FBS-free media (Figure 3), it was unclear whether viable and functional cells could be maintained in FBS-free medium over this same time period. Studies to evaluate this issue revealed that over a 4 h period, RAW264.7 cells in DMEM demonstrated essentially identical viability (Figure 4A), cell cycle progression (Figure 4B), and metabolic activity (Figure 4C) in the absence or presence of FBS (10%). Even after 24 h, the viability (Figure 4A) and cell cycle profiles (Figure 4B) were not significantly different for RAW264.7 cells cultured in the absence or presence of FBS. The metabolic activity of RAW264.7 cells increased after 24 h, but significantly more so in the presence than absence of FBS (Figure 4C), which we speculate was due to greater overall proliferation and number of cells in FBS-enriched medium. These results confirmed that, for at least 4 h, *in vitro *models of infection can be conducted under entirely non-germinating culture conditions without loss of host cell viability, cell cycle progression, or metabolic function.

### Germination state of spores does not alter the uptake by mammalian cells

**Figure 5:**
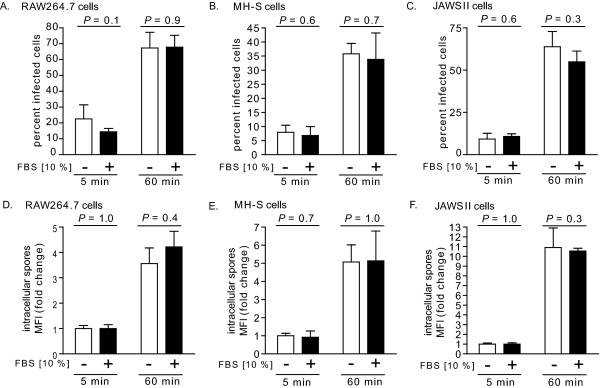
**Uptake of *B. anthracis *spores into mammalian cells cultured under germinating or non-germinating conditions**. RAW264.7 cells (A, D), MH-S cells (B, E), or JAWSII cells (C, F) were incubated with *B. anthracis *spores (MOI 10) in DMEM, RPMI, or DMEM, respectively, in the presence (+, black bars) or absence (-, white bars) of FBS (10%), and then evaluated at 5 or 60 min by flow cytometry and in the presence of trypan blue (0.5%) for the percentage of cells with intracellular spores (A-C), and, for total cell associated spore fluorescence (D-F), as described under Materials and Methods. (A-C) The data are rendered as the percentage of infected cells with the entire population that has internalized spores. (D-F) The data are expressed as the change in MFI, normalized to cells at 5 min post infection in FBS-free medium. To generate the bar graphs, data were combined from three independent experiments, each conducted in triplicate. Error bars indicate standard deviations. The *P *values were calculated to evaluate the statistical significance of the differences in percent infected cells (A) or total intracellular spores (B) between cells incubated in the absence or presence of FBS.

The demonstration that cultured RAW264.7 cells remained viable and functional in FBS-free cell culture medium did not directly address the possibility that spore uptake by mammalian cells might be substantially different under germinating and non-germinating cell culture conditions. To evaluate this issue, Alexa Fluor 488-labeled spores were incubated with RAW264.7, MH-S, or JAWSII cells (MOI 10) in the absence or presence of FBS (10%). After 5 or 60 min, intracellular spores were monitored using flow cytometry to measure cell associated fluorescence that was not sensitive to the membrane-impermeable, Alexa Fluor 488 quenching agent, trypan blue [46]. These studies revealed that for each cell line tested, neither the percentage of infected cells within the population (Figure 5A-C), nor the overall increase in intracellular spores (Figure 5D-F), was significantly different in the presence or the absence of FBS. Collectively, these results revealed that the uptake *of B. anthracis *spores by mammalian cells is essentially the same within germinating and non-germinating *in vitro *environments.

### Germination state of spores influences the number of viable, intracellular *B. anthracis*

**Figure 6:**
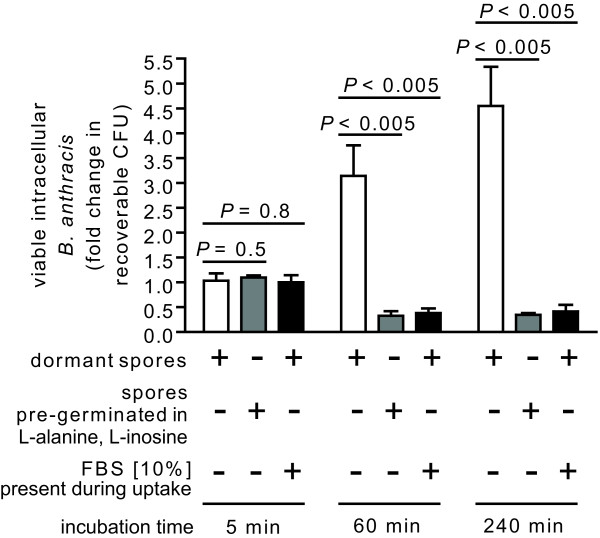
**The germination state of spores influences the viability of intracellular *B. anthracis***. RAW264.7 cells were incubated for 30 min with dormant *B. anthracis *spores (MOI 10) in DMEM in the presence (+, black bars) or absence (-, white bars) of FBS (10%), or, with pre-germinated spores (MOI 10) in DMEM in the absence of FBS (grey bars). *B. anthracis *spores were pre-germinated by incubation for 30 min in PBS pH 7.2 supplemented with L-alanine and L-inosine (both at 10 mM), and then washed twice with PBS pH 7.2 to remove germinants. After 30 min, the cells were washed to remove extracellular *B. anthracis*, and then further incubated with FBS (10%) and, as described under "Methods," with gentamicin to germinate and kill any remaining spores that had not been germinated. After 15 min, the cells were washed and then further incubated in the absence of gentamicin. At 5, 60, or 240 min after removal of gentamicin, as indicated, the RAW264.7 cells were lysed, and the lysates were evaluated for viable *B. anthracis*, as described under Materials and Methods. The data were rendered as the fold-change in recoverable CFU in the absence or presence of FBS, relative to cells at 5 min post infection in the absence of FBS. The rendered data were combined from three independent experiments, each conducted in triplicate. Error bars indicate standard deviations. The *P *values were calculated to evaluate the statistical significance of the differences in recoverable CFU between cells infected in the absence or presence of FBS.

Although the uptake of *B. anthracis *spores into mammalian cells was independent of the presence or absence of FBS in the culture medium, it was not clear whether the outcome of infection would also be similar under germinating and non-germinating conditions. To evaluate this issue, the recovery of viable, intracellular *B. anthracis *was compared subsequent to uptake by RAW264.7 cells in the absence or presence of FBS (10%), using the gentamicin protection assay [11,21,46,47]. These studies indicated that there were not significant differences in intracellular CFU after 5 min post-infection (Figure 6). However, after 60 or 240 min post infection, significantly greater CFU were recovered from cells in DMEM lacking FBS relative to cells incubated in the presence of FBS (Figure 6). To evaluate whether these differences might be attributed strictly to the presence or absence of FBS, similar studies were conducted in the absence of FBS, however this time using spores that had been pre-germinated for 30 min with DMEM supplemented with L-alanine/L-inosine (both at 10 mM). Similar to spore uptake in the presence of FBS, significantly fewer CFU were recovered from cells incubated with pre-germinated spores in the absence of FBS relative to cells incubated with dormant spores in DMEM lacking FBS (Figure 6). These results support the idea that the germination state of spores, rather than the presence or absence of FBS, is an important determinant of the outcome of infection. Similar results were obtained when studies were conducted with MH-S cells and JAWSII cells (not shown). Although the reasons underlying the greater recovery of spores from infections conducted under non-germinating conditions are not clear, we speculate that germinated spores might be more susceptible than dormant spores to killing after uptake from the cell surface. This potential explanation is consistent with earlier reports that spores that had been intentionally pre-germinated prior to exposure to mammalian cells were more readily killed than dormant spores upon uptake into mammalian cells [20,22]. These results support the idea that the germination state of *B. anthracis *spores is a critical determinant of the fate of the intracellular bacteria.

### Germination state of *B. anthracis *spores influences the viability of RAW264.7 cells during *in vitro *infection

**Figure 7:**
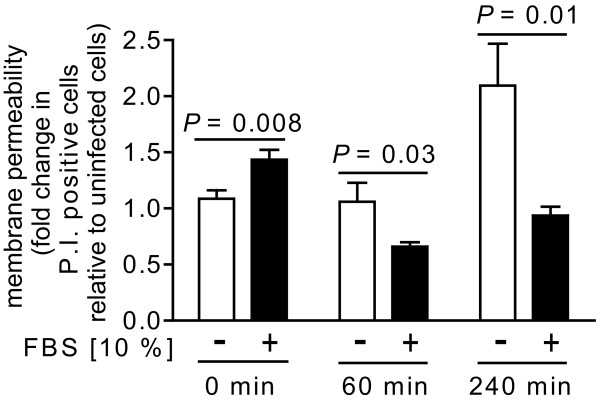
**The germination state of spores influences the viability of *B. anthracis*-infected cells**. RAW264.7 cells were incubated for 30 min with *B. anthracis *spores (MOI 10) in DMEM in the presence (+, black bars) or absence (-, white bars) of FBS (10%). After 30 min, the cells were washed to remove extracellular *B. anthracis*, and then further incubated with FBS (10%) and, as described under "Methods," with gentamicin to germinate and kill any remaining spores that had not been germinated. After 15 min, the cells were washed and then further incubated in the absence of gentamicin. At 0 (immediately after gentamicin removal), 60, or 240 min after removal of gentamicin, as indicated, the cells were evaluated for mammalian cell death via PI uptake, as described under Materials and Methods. The data are rendered as the fold-increase of PI uptake relative to non-infected cells in the absence or presence of FBS at 5, 60, or 240 min, as indicated. The rendered data have been combined from three independent experiments, each conducted in triplicate. Error bars indicate standard deviations. The *P *values were calculated to evaluate the statistical significance of the differences between the fold-increase of PI uptake between cells incubated with spores in the absence or presence of FBS.

The greater number of viable, intracellular *B. anthracis *recovered from cells infected under non-germinating conditions (Figure 6) prompted us to examine whether the viability of infected host cells might also be influenced by the germination state of spores during uptake. To evaluate this issue, RAW264.7 cells were incubated with *B. anthracis *spores (MOI 10) in the presence or absence of FBS (10%). Subsequent to employing the same gentamicin-protection procedure used for monitoring intracellular *B. anthracis *(Figure 6), PI uptake by RAW264.7 cells was measured at 5 min, 1 h, and 4 h, post-infection. These studies revealed that at 4 h post-infection, there was approximately 2-fold greater PI uptake, indicating a significantly greater loss in viability of RAW264.7 cells that had been incubated with spores in FBS-deficient medium, as compared to FBS-enriched medium (Figure 7). When evaluated at 8 h post-infection, PI uptake was nearly 5-fold greater in RAW264.7 cells that had been incubated with *B. anthracis *spores in FBS-deficient medium (data not shown). Understanding the reasons underlying these significant differences in the viability of infected cells will require future studies, but we speculate that the greater intracellular load of *B. anthracis *in cells infected under non-germinating conditions (Figure 6) may directly contribute to the higher degree of cell death.

### The importance of culture medium during *in vitro *infection models

Despite compelling evidence that during *in vivo *infection, the alveolar spaces of the lungs are intrinsically non-germinating, and dormant spores are taken up by mammalian cells prior to germination [5-7,23-27], many studies involving *in vitro *models of infection have been conducted under germinating medium conditions [20,28-34]. Most studies have been conducted in cell culture medium containing 2-10% FBS, including those using RAW264.7 cells [48,49], and the germination state of spores have not generally monitored or controlled for during *in vitro *infections.

Several *in vitro *models have employed additives to the culture medium in an attempt to modulate germination. Several studies used D-alanine and/or D-histidine [22,32,50], known inhibitors of germination initiation. However, interpretation of these studies may be complicated by the finding that D-alanine/D-histidine, when added subsequent to spore uptake into macrophages, alter the extent to which spores germinate [32], suggesting that these D-amino acid germination inhibitors diffuse into host cells and affect spore germination within intracellular vesicles. Horse serum has been used by several groups to limit spore outgrowth during infection [20,32,33,51]. However, 10% horse serum in DMEM only slows, but does not eliminate the germination initiation of spores [20]. The finding that RAW264.7 cells maintain viability, cell cycle progression, and mitochondrial metabolic activity for at least 4 h when maintained in serum-free medium (Figure 4), indicate that *in vitro *infections, at least with RAW264.7 cells, can be conducted under non-germinating conditions using FBS-free medium.

### The outcome of infection is influenced by the germination state of spores

Both spore (Figure 6) and host cell (Figure 7) viability were influenced by the germination state of spores at the time of uptake. Because several cell lines internalized the same number of spores under both germinating and non-germinating conditions (Figure 5), it is unlikely that differences in the outcome of infection are due solely to initial differences in spore load. Rather, we speculate that, in contrast to dormant spores, germinated spores might be more vulnerable to growth inhibition and/or killing during phagocytosis. These results are consistent with previous reports that when infections were conducted with spores in medium containing FBS or fetal calf serum (e.g. germinating conditions), there were generally, within the first 4-5 h post-infection, losses in intracellular CFU recovered from primary human dendritic cells [17], primary mouse alveolar macrophages [17], J774.A1 murine macrophage-like cells [18], bone marrow derived macrophages from A/J mice [34], or RAW 264.7 cells [13].

## Conclusions

This study demonstrates that the infection of RAW 264.7 cells by *B. anthracis *spores is influenced by the germination state of spores, as dictated by the *in vitro *culture medium. The extent to which the germination state of *B. anthracis *spores ultimately affects the outcome of infections using cells other than RAW264.7 cells may ultimately depend on the properties idiosyncratic to that particular cell type or cell line. However, our results indicate the importance of rigorously considering the germinating properties of the culture medium when establishing *in vitro *models to study the infection of host cells with *B. anthracis *spores.

## Methods

### Spore preparations and fluorescent labeling

Spores were prepared from *B. anthracis *Sterne 7702 and enumerated using a hemacytometer (Thermo Fisher Scientific, Waltham, MA), as described previously [46]. As quality control, spore preparations were tested for both heat resistance and the capacity to germinate, as described [46].

### Mammalian cell culture

Abelson murine leukemia virus-transformed murine macrophages derived from ascites of BALB/c mice (RAW 264.7 macrophage-like cells; CRL-2278; ATCC, Manassas, VA) were maintained within a humidified environment at 37°C and under 5% CO_2 _in complete DMEM, (Thermo Scientific, Waltham, MA) containing penicillin (100 U; Gibco BRL, Grand Island, NY), streptomycin (0.1 mg/ml; Gibco BRL), L-glutamine (2 mM; Sigma, St. Louis, MO), and FBS (10%; JRH Biosciences, Lenexa, KS). MH-S cells (CRL-2019; ATCC) were maintained within a humidified environment at 37°C and under 5% CO_2 _in complete RPMI medium (Thermo Scientific) containing penicillin-streptomycin (100 U, Gibco BRL), L-glutamine (4 mM), and FBS (10%). JAWSII (CRL-11904; ATCC) were maintained within a humidified environment at 37°C and under 5% CO_2 _in complete MEMα (Thermo Scientific) containing penicillin-streptomycin (100 U), L-glutamine (4 mM), and FBS (20%). All tissue culture plasticware was purchased from Corning Incorporated (Corning, NY).

### Evaluation of *B. anthracis *spore germination in cell culture media

Using 96 well plates, spores prepared from *B. anthracis *7702 (1.0 × 10^8 ^spores/mL) were incubated at 37°C and under 5% CO_2 _in BHI (BD Biosciences, San Jose, CA), LB (0.1% tryptone, BD Biosciences; 0.05% yeast extract, BD Biosciences; 0.05% NaCl, Fisher Chemical, Fairlawn, NJ), PBS pH 7.2 (Mediatech, Manassas, VA), or germinating amino acids (10 mM L-alanine, 10 mM L-inosine, both from Sigma) in PBS pH 7.2. In other studies, spores were incubated in 96 well plates (10^8 ^spores/mL) and at 37°C and under 5% CO_2 _in the following cell culture media without or with FBS (10%, unless otherwise indicated; Mediatech): DMEM (0.1, 0.5, 1, 5 or 10% FBS), RPMI-1640, MEMα modification (10 or 20% FBS), MEM (Mediatech), AMEM (Gibco), EMEM (Mediatech), BME (Sigma), CIM (Gibco), Ham's F-12 (Mediatech), McCoy's 5A (M5A, ATCC), or DMEM with 10% FBS and 10 mM D-alanine (Sigma) and D-histidine (Sigma). In some assays, FBS obtained from Mediatech was substituted with FBS purchased from Invitrogen or Sigma. As described previously [39], spore germination was evaluated by measuring loss in spore refractility or loss of heat resistance, while outgrowth was monitored by monitoring the elongation of bacilli using a Delta Vision RT microscope (Applied Precision; Issaquah, WA), outfitted with an Olympus Plan Apo 100 × oil objective. DIC images were collected using a Photometrics CoolSnap HQ camera; (Photometrics, Tucson; AZ), and processed using SoftWoRX Explorer Suite (version 3.5.1, Applied Precision Inc).

### Pre-conditioning of cell culture media

To pre-condition cell culture medium, monolayers of RAW264.7 or MH-S cells in 24-well plates (80 to 95% confluency) were washed three times with Hanks' balanced salt solution (HBSS) and then incubated in DMEM (for RAW264.7 cells) or RPMI-1640 (for MH-S cells) without FBS and penicillin-streptomycin in a humidified environment at 37°C and under 5% CO_2_. After 4 or 24 h, the medium was withdrawn, centrifuged (600 × *g *for 5 min), and the supernatant was filter sterilized using a 0.22 μm filter (Corning). To evaluate heat sensitivity, some of the filter-sterilized pre-conditioned medium was incubated at 95°C for 10 min or, alternatively, 65°C for 30 min Alternatively, some of the filter-sterilized pre-conditioned medium (3 mL) was dialyzed four times against PBS pH 7.2 (500 mL), using dialysis tubing with 12,000-14,000 molecular mass cutoff (Spectrum Laboratories, Inc., Rancho Dominguez, CA), each time for 6 h.

### Mammalian cell viability

To evaluate the viability of RAW264.7, MH-S, or JAWSII cells, alterations in membrane permeability, as indicated by relative PI (1 μg/mL; Invitrogen Molecular Probes, Eugene, OR) uptake, were measured using flow cytometry, as previously described [46].

### Flow cytometry

Analytical flow cytometry was carried out using a Beckman Coulter EPICS XL-MCL™ flow cytometer equipped with a 70-μm nozzle, 488 nm line of an air-cooled argon-ion laser, and 400 mV output. The band pass filter used for detection of Alexa Fluor 488 spores was 525/10 nm. The long pass filter used for cell cycle phase determination assays and mammalian cell viability assays was 655 nm/LP. Cell analysis was standardized for side/forward scatter and fluorescence by using a suspension of fluorescent beads (Beckman Coulter Inc., Fullerton, CA). At least 10,000 events were detected for each experiment (>2000 events per min). Events were recorded on a log fluorescence scale and evaluated using FCS Express 3.00.0311 V Lite Standalone. Sample debris (as indicated by lower forward and side scatter and a lack of PI staining) represented a small fraction (1 to 2%) of the detected events and was excluded from analysis.

### Cell cycle assay

To compare the cell-cycle profiles of RAW264.7 cells cultured in FBS-containing medium or FBS-free medium, relative PI uptake was measured using flow cytometry. At 4 or 24 h, as indicated, cells were incubated at room temperature with Cellstripper™ (Mediatech). After 15 min, the cells were further diluted with PBS pH 7.2 containing 10% FBS (800 mL). The cell suspensions were centrifuged for 5 min at 500 × *g *at room temperature. The pellets were resuspended in 300 μL of PBS pH 7.2 at room temperature, fixed by adding anhydrous ethanol (100%, 700 μL prechilled to -20°C, Fisher Scientific) with continuous vortexing, and then further incubated for at least 2 h at -20°C. The cells were centrifuged for 5 min at 500 × *g *at room temperature, and the pellets were resuspended in 1 mL of PBS pH 7.2, and then incubated at room temperature for 30 min. The cells were centrifuged 5 min at 500 × *g *at room temperature. The cell pellets were resuspended in 300 μL PBS pH 7.2, 0.1% Triton X-100 (MP Biomedicals, Solon, OH), DNase-free RNase A (100 mg/mL; Sigma), and PI (10 μg/mL), and further incubated at room temperature for 60 min. The stained cells were analyzed by flow cytometry.

### Mammalian cell metabolism assay

To compare the metabolic activities of RAW264.7 cells cultured in FBS-containing medium or FBS-free medium, the relative conversion of tetrazolium 3-(4,5-di-methylthiazol-2-yl)-2,5-diphenyltetrazolium bromide (tetrazolium; 5 mg/mL, Sigma) to formazan over 30 min and at 37°C was measured at 570 nm with a Synergy 2 plate reader (BioTek Instruments, Inc., Winooski, VT), as described [39,52].

### *In vitro *infection of mammalian cells with *B. anthracis*

Mammalian cells (5.0 × 10^5 ^total cells/well) were incubated in the appropriate complete medium, as indicated above under "Mammalian cell culture," for two days in a humidified environment at 37°C and under 5% CO_2_, resulting in 80-95% confluency. To calculate the number of spores needed to achieve MOI 10, cells from several wells were detached using Cellstripper™ and enumerated using a hemacytometer. The cells were used only if greater than 90% of the cells excluded trypan blue; generally, greater than 95% of the cells within the monolayer excluded trypan blue. Prior to the addition of labeled spores, cells were washed three times with HBSS and then incubated in DMEM (RAW264.7 and JAWSII) or RPMI-1640 (MH-S), without or with FBS, as indicated. To synchronize the exposure of cells to spores, spores were immediately and gently centrifuged (600 × *g *for 5 min) onto the surfaces of cells. The plates were incubated within a humidified environment at 37°C and under 5% CO_2 _for the indicated times prior to analysis.

### Quantification of *B. anthracis *uptake by mammalian cells

Internalization of *B. anthracis *spores by mammalian cells was quantified using a previously described flow cytometry based assay [46]. Briefly, the indicated mammalian cell lines were seeded into 48-well plates (Corning) in order to achieve 80-95% confluency after two days of incubation. As previously described [46], *B. anthracis *spores were labeled using an amine reactive Alexa Fluor^® ^488 carboxylic acid, succinimidyl ester (Molecular Probes-Invitrogen). Alexa Fluor 488-labeled *B. anthracis *spores were quantified using a hemacytometer, added to cells at the desired MOI, and immediately but gently centrifuged (300 *xg *for 5 min) onto the surface of cells. The plates were incubated within a humidified environment at 37°C and under 5% CO_2 _for the indicated times prior to analysis using flow cytometry, as previously described [46]

To discriminate intracellular spores from those which remain surface-associated during infection, cells were analyzed in the presence of trypan blue, a membrane-impermeable, Alexa Fluor 488^® ^fluorescence quenching agent [53]. Previously, 0.5% trypan blue was demonstrated to completely quench the fluorescence emission of Alexa Fluor 488-labeled spores bound to the surface of mammalian cells, while having no affect the fluorescence emission of internalized spores [46]. From these data, the percentage of cells with internalized *B. anthracis *was calculated by dividing the number of viable cells with greater than background auto-fluorescence by the total number of viable cells. For spore internalization experiments, viable mammalian cells (typically 90-98% of the total events) were readily identified by their high forward scatter and lack of propidium iodide (PI) staining. A second distinct population, (2-10%) of dead cells was routinely detected with relatively lower forward scatter (which indicates a smaller size) and positive PI staining (indicating non-viable cells; data not shown). Over the course of 60 min, we observed no detectable increase in cell death in the presence of labeled spores, as indicated by PI uptake (data not shown). Finally, sample debris (as indicated by relatively lower forward and side scatter and a lack of PI staining) represented a small fraction (1-2%) of the detected events. Based on these data, the data from subsequent experiments were gated to include only viable cells, while excluding non-viable cells, cellular debris, and spores not associated with cells. Alternatively, the time dependent total uptake of spores was determined by plotting the geometric mean of the fluorescence intensity (MFI).

### Quantification of viable, intracellular *B. anthracis*

Cells were incubated with dormant *B. anthracis *spores, as indicated above. For germinated *B. anthracis *spore infections, *B. anthracis *spore were germinated with 10 mM L-alanine and L-inosine in 1 × PBS pH 7.2 for 30 min and washed twice with 1 × PBS pH 7.2 to remove germinants and enumerated as described above. After 30 min, cells were washed three times with HBSS, and further incubated in the indicated medium with FBS (10%) and gentamicin (100 μg/ml) to kill all external germinated spores. After 15 min, the cells were washed three times with HBSS, and further incubated in the indicated appropriate medium supplemented with FBS (10%). At the indicated times, the cells were lysed by incubating with sterile tissue culture grade water (Mediatech) for 5 min at 25°C. Serial dilutions of the lysates were plated on LB agar plates and incubated overnight at 37°C. CFU were enumerated by direct counting of visible colonies and correcting for the appropriate dilution.

### Statistics

All data are representative of those from three or more independent experiments. The *Q*-test was performed to eliminate data that were statistical outliers [54]. Error bars represent standard deviations. *P *values were calculated with Student's *t *test using paired, one-tailed distribution. *P *< 0.05 indicates statistical significance. Statistical analyses to calculate means, standard deviations, and Student's *t *tests, were calculated using Microsoft Excel (version 11.0).

## Authors' contributions

IG assisted in experimental design, carried out the experiments, analyzed data, and drafted the manuscript. TB assisted in experimental design and data analysis, carried out the experiments, and assisted in drafting the manuscript. AP and BS conceived the study and performed preliminary experiments. SC carried out experiments. WV helped to draft the manuscript. SB assisted in experimental design and drafted the manuscript. All authors read and approved the final manuscript.
